# Laryngology

**Published:** 1893-09

**Authors:** B. J. Baron


### LARYNGOLOGY.

Hobbs, in a paper entitled "The Uses and Abuses of Cocaine,"[1] gives several useful hints about this drug which are worthy of note. The softer the mucous tissue to which it is applied, the sooner is its effect reached, and the less the quantity necessary; and as a consequence, the quicker and more decided is the toxic effect when its application is persisted in to this extent. The area of the mucous membrane involved determines the general effect rather than the strength of the solution that is used. Hence sprays of a weak solution freely employed are liable to produce toxic symptoms, when much stronger solutions can be applied with a wad to a limited area, almost with impunity.

Idiosyncrasy in the matter of the strength of the solution, of the time that it takes to affect the mucous membrane locally, and whether or not it affects the system generally, ought never to be forgotten. The adage, "What is one man's meat is another's poison," is apt to prove its truth very disagreeably to careless users of cocaine.

If we desire to affect a small piece of mucous membrane, as in galvano-cautery operations, a 10 to 20 per cent. solution should be used, and the effect may be expected in from four to eight minutes. If, on the other hand, we want to reduce the general turgescence of a mucous surface, a spray of one or two per cent. is usually efficacious in one or two minutes.

The writer mentions two cases of priapism which had resisted treatment by the ordinary methods, which had been continuously employed for 24 hours in one case and 36 hours in the other; these were completely and permanently cured in ten minutes after spraying cocaine camphor menthol vaseline on the interior of the nose. This only confirms what many of us have noticed; viz., that there is a relation between the erectile tissue of the turbinates and of the genital organs.

It is strongly recommended as a hæmostatic before the removal of the tonsil with the guillotine. Out of 4,000 cases of tonsillotomy in which it was employed, either painted on the tonsil or injected by means of a hypodermic syringe, before operation, not one case gave the operator any anxiety on the score of hemorrhage.

The greatest amount of abuse comes from the frequent use of sprays, and especially in hay-fever cases. Physicians are said to provide most of the cocaine *habitués*.

Bryson Delavan[2] calls attention to the importance of discovering, before operations on the throat, the possible existence of a diathetic condition which might influence the result of the operation. In cases of exophthalmic goître and general

[1] *Journ. Laryn., Rhin., and Otol.*, Nov., 1892. [2] *Ibid*, p. 538.

lymphadenoma, he concludes that the removal of enlarged tonsils and adenoid hypertrophy in the vault of the pharynx is attended by no extraordinary risk. Complete removal of the offending tissue is more difficult in strumous cases, recovery is slower, and recurrence more probable. Rheumatic patients present no especial difficulties, and the prognosis is very good. Hypertrophy of the pharyngeal tonsil is unusually common in hæmophilic subjects; the only known accidents that have occurred in America, after its removal, have been in these patients—and there is no safe plan of operation.

Booker, Koplik, and Lewis Smith [1] have all discussed the question of the accurate diagnosis of diphtheria and pseudo-diphtheria, and their conclusion appears to be that the Loeffler bacillus is found in the throat of almost every patient suffering from true diphtheria, whether membranous or not. Membrane is not considered pathognomonic of diphtheria (Baginsky, Hoffmann, Escherich, and others are quoted in support of this opinion). Streptococci were found in all Booker's cases of pseudo-diphtheritic angina, staphylococcus aureus occurred in 21 cases of scarlatina and 3 cases of measles, and staphylococcus albus in 4 cases of scarlatina: no Loeffler's bacillus was found in these cases

Follicular appearances on one tonsil may be accompanied by true diphtheria and a small patch of membrane on the other side. A coating of yellow exudate, or specks of it, may exist in which Loeffler's bacilli are found. In others there are nothing but streptococci, and then the cases resemble a simple tonsillitis.

The Loeffler bacillus has been found on the inflamed pharyngeal mucous membrane, which became coated with fibrinous membrane; but at no time was there a picture of diphtheria. Martin [2] analysed 200 cases, supposed to be diphtheritic. In 72 (of which 29 were croupous) the Loeffler bacillus was absent. Exposure to scarlatina and measles had occurred in some of these cases. The mortality was largest in cases of true diphtheria, *i.e.* when the Klebs-Loeffler bacillus was present. A pseudo-membranous inflammation appears possible when only cocci are present, when no toxine like that produced by the true Loeffler bacillus exists.

Henoch, Wurtz, and Bourges regard the scarlatinal necrosis as due to the intensity of the inflammation. When it is truly diphtheritic it occurs late in scarlet fever. The streptococcus, the supposed agent of pseudo-diphtheria, is present in this necrosis, but the Loeffler bacillus is rarely found.

Ashby [3] describes a ready method for the detection of the Loeffler bacillus. Detach a piece of membrane, place it for five

[1] *Arch. of Pediatrics*, Sept., 1892. (Abs in *Journ. Laryn., Rhin., and Otol.*, Nov., 1892.)

[2] *Ann. de l'Inst. Pasteur*, May, 1892. [3] *Brit. Med. Journ.*, Feb. 6, 1892, p. 276.

minutes in a 20 per cent. solution of boracic acid, draw it along the free surface of sterilised blood serum in a test tube, which is maintained at a temperature of 37°C. for from 12 to 24 hours, when small round colonies of the culture are visible, and are to be stained in carbol fuchsine.

In a paper which he read in the section of Public Medicine at the Newcastle meeting of the British Medical Association, Eustace Hill[1] drew attention to cases of infectious sore throat which are neither diphtheritic nor scarlatinal, in which the appearances are those of the so-called "follicular tonsillitis," fever and prostration accompanying them, and in which neither membrane nor cutaneous rash nor desquamation makes its appearance. The mortality was much lower, and the ages of those attacked higher than is the case in true diphtheria. Infection was transmitted through the milk supply; and isolation is imperatively necessary.

Our experience undoubtedly teaches us that it is very difficult to exclude the possibility of a spotted, septic throat being diphtheritic; and when one sees that the trend of scientific opinion is that only the skilled expert bacteriologist can definitely settle the point, it is our duty to isolate as far as possible all cases of "acute follicular tonsillitis," and patients in whom septic phlegmonous uvulitis and pharyngitis makes its appearance. This has been my own practice for years past.

Dunn[2] draws attention to five cases of "pin sensation in the throat;" two of these were men and three young women. In my own experience, such patients are usually considered to be hysterical, and nothing of any use is even attempted to be done for them. This opinion is erroneous, and is based on inefficient examination, since acutely inflamed granulations behind the posterior pillar of the fauces on the side where the pain is felt are commonly present, and accurate galvano-cauterisation of these cures it.

Spencer Watson[3] alludes to the influence of nasal stenosis on the general health; and *early* removal of this impediment to nasal breathing, whether due to obstruction in or behind the notrils, is insisted on. All nasal specialists know how, spite of the voluminous writing on this matter, children are allowed to acquire and keep up mouth breathing, with all the attendant asthma, diurnal and nocturnal throat cough, deafness, and dulness of intellect for years, on the plea that they will grow out of it, when all the time they are more and more growing confirmed in it. And all for the want of a timely small operation.

Heryng,[4] writing on the surgical treatment of laryngeal

[1] *Brit. Med. Journ.*, Aug. 19, 1893.

[2] *New York Med. Journ.*, June 11, 1892. [3] *Lancet*, Sept. 10, 1892.

[4] *Journ. Laryn., Rhin., and Otol.*, Aug., 1893.

phthisis, says: "To-day we can say with genuine pleasure that the harmful theory of the incurability of laryngeal phthisis is exploded, and that it is now a thing of the past. Even the most severe cases, presenting a high degree of infiltration of the epiglottis, and accompanied by tubercular infiltration of the ventricular band and cartilage of Santorini, and by deep ulcers of the posterior laryngeal wall, can, nevertheless, be cured. Further, that they can remain so for some years without recurrence, as is evident from the cicatrices in spite of advancing pulmonary phthisis." He also believes that tubercle attacks the larynx primarily, and goes on to affect the lung secondarily, and that this infection may be prevented by adequate surgical measures. These consist in curettement and the thorough application of lactic acid; the reaction from this, whilst it is well marked, is rarely serious. Galvano-cautery produces more reaction; electrolysis is slower, but is efficient, although less so than the acid treatment. I have seen tubercular pharyngeal ulcers heal after thorough scraping and firm application of pure lactic acid.

B. J. BARON.

